# Supplementary material for: Transitions in cell potency during early mouse development are driven by Notch
Source: eLife. 2019 Apr 8;8:e42930. doi: 10.7554/eLife.42930 (PMC6486152; doi:10.7554/eLife.42930)
Supplement: Supplementary file 1. [file elife-42930-supp1.docx]

| Gene | Primer F | Primer R |
| --- | --- | --- |
| *Actin* | CAGAAGGAGATTACTGCTCTGGCT | TACTCCTGCTTGCTGATCCACATC |
| *18S rRNA* | GCAATTATTCCCCATGAACG | GGCCTCACTAAACCATCCAA |
| *Cdx2* | TCAACCTCGCCACAACCTTCCC | TGGCTCAGCCTGGGATTGCT |
| *Gata3* | GGGTTCGGATGTAAGTCGAG | CCACAGTGGGGTAGAGGTTG |
| *Oct4* | ATCAGCTTGGGCTAGAGAAGGATG | AAAGGTGTCCCTGTAGCCTCATAC |
| *Nanog* | CTTACAAGGGTCTGCTACTGAGATGC | TGCTTCCTGGCAAGGACCTT |
| *Tle4* | CTGGACAGGTGGTTTGGACAA | GAGGTGAAGTCATGTTGCTGC |
| *Tbx3* | TGAGGTGCTCTGGACTGGAT | ACCATCCACCGAGAGTTGTG |
| *Prdm14* | CAACCTTGGAAACTGGCAGC | AAGCATCAAGAGGGGCCATC |
| *Dppa3* | GACCCAATGAAGGACCCTGAA | GCTTGACACCGGGGTTTAG |
| *Esrrb* | GGACACACTGCTTTGAAGCA | ACAGATGTCTCTCATCTGGC |
| *Fgf5* | AAGTAGCGCGACGTTTTCTTC | CTGGAAACTGCTATGTTCCGAG |
| *Pou3f1* | TCGAGGTGGGTGTCAAAGG | GGCGCATAAACGTCGTCCA |
| *Otx2* | TATCTAAAGCAACCGCCTTACG | AAGTCCATACCCGAAGTGGTC |
| *Eomes* | TTCACCTTCTCAGAGACACAGTTCAT | GAGTTAACCTGTCATTTTCTGAAGCC |
| *Gata6* | TCATTACCTGTGCAATGCATGCGG | ACGCCATAAGGTAGTGGTTGTGGT |
